# Characteristics of Filter Cake Exfoliation in Upward Ultrafiltration of Nanoparticle Suspensions

**DOI:** 10.3390/membranes1010059

**Published:** 2011-01-20

**Authors:** Yasuhito Mukai, Aya Nishio

**Affiliations:** Department of Chemical Engineering, Nagoya University, Furo-cho, Chikusa-ku, Nagoya 464-8603, Japan; E-Mail: myosotis_scorpioides@softbank.ne.jp

**Keywords:** nanoparticle, ultrafiltration, filter cake exfoliation, steady filtration rate, binary mixture

## Abstract

Downward and upward ultrafiltration (UF) was performed using the suspensions of nanosized colloidal silica with different particle diameters and their filtration rates were compared. In downward UF, the filtration rate decreases as the particle diameter decreases because the specific filtration resistance of the filter cake becomes significantly higher. In contrast, the filtration rate in upward UF increases with the decrease in the particle diameter because the filter cake consisting of small particles is exfoliated much more easily under the influence of gravity than that of large ones. In order to evaluate the characteristics of the filter cake exfoliation, the steady filtration rate in the upward mode was measured. The steady filtration rate has a tendency to decrease with particle concentration as well as mean particle diameter. Therefore, when the small particles are added into a given concentration of large particle suspension, the mean particle diameter decreases and the total particle concentration increases due to the dosage of small particles. This results in a maximum of the steady filtration rate at a certain dosage of small particles. Moreover, an estimation equation was proposed for predicting the steady filtration rate in upward UF of colloidal silica suspensions with various mean particle diameters and total particle concentrations.

## 1. Introduction

Recently, many technologies associated with nanotechnology are being developed and applied to widely diversified fields. For example, the development of technologies to synthesize and functionalize nanoparticles is being promoted with the aim of applications for chemical industry, bioindustry, medical technology, electronics and energy. Also, studies on the processing of nanoparticles, such as the development of advanced separation process, are of increasing importance with the progress of nanotechnology. Especially, membrane separation including ultrafiltration (UF) is one of the most promising technologies and the establishment of technology and theory about membrane separation of nanoparticles is urgently needed. However, while many researchers have reported microfiltration of submicron particle suspensions and UF of protein solutions, only minor attention has been given to studies on membrane filtration of nanoparticle suspensions [1,2].

In a previous study [2], the flux behaviors and the filter cake properties were explored in UF experiments of nanoparticle suspensions. As a result, it was unequivocally demonstrated that the nanoparticle cake formed in upward UF, in which the filtrate flow was in an upward direction, was characterized by high flowability and had a tendency to exfoliate under the influence of gravity. A similar behavior was also observed in upward UF of proteinaceous solutions [3]. This unique characteristic of nanoparticle cake is of great interest to both industry and academia, but the effects of various dominant factors on cake exfoliation characteristics are poorly understood.

In this study, upward UF experiments of nanosized silica sol were performed at various conditions of particle diameter and concentration. The primary concern is attempting to provide a fundamental understanding of exfoliation characteristics of the filter cake consisting of single or binary nanoparticles.

## 2. Experimental Methods and Materials

**Figure 1:**
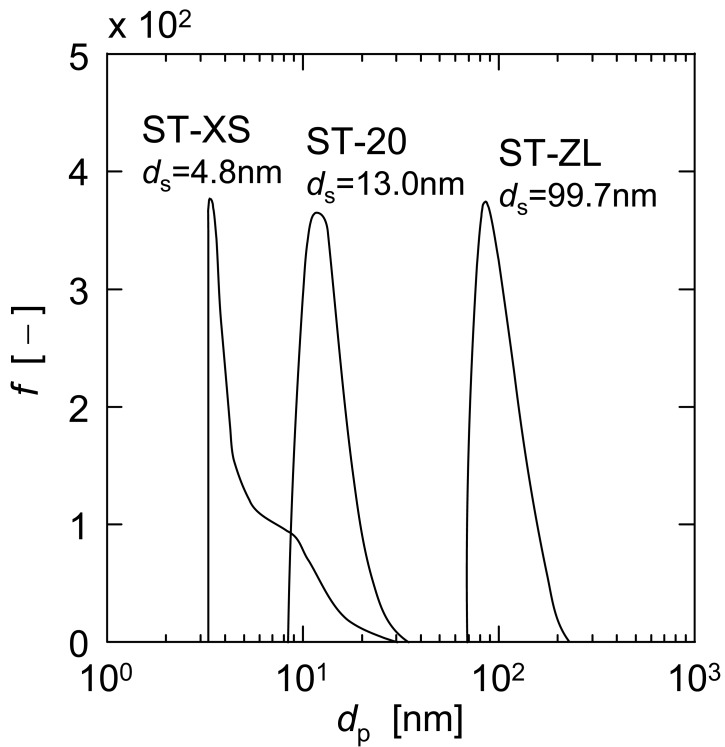
Size distributions of nanoparticles.

The nanoparticles used in the UF experiments were three kinds of colloidal silica with different particle diameters, ST-XS, ST-20 and ST-ZL, supplied by Nissan Chemical Industries. The mass-based size distributions of three nanoparticles measured using a dynamic light scattering spectrometer (DLS-7000, Otsuka Electronics Co.) are shown in Figure 1. The distributions are all relatively sharp and the surface mean diameters *d*_s_ of ST-XS, ST-20 and ST-ZL are 4.8, 13.0 and 99.7 nm, respectively. The single component or binary suspensions of these particles with various concentrations were prepared by suspending them in the ultrapure, deionized water.

**Figure 2:**
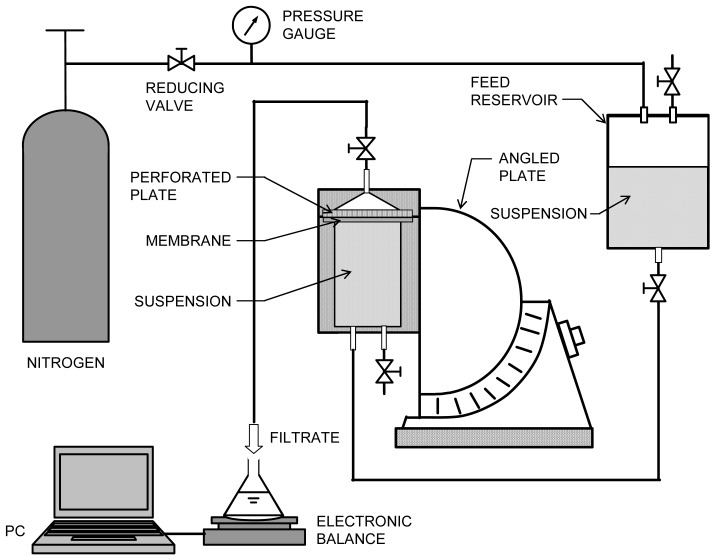
Schematic diagram of upward UF apparatus.

The UF experiments were carried out by means of two modes of downward filtration in which the filtrate flow is in the same direction as gravity, and the upward one where the filtrate flow is opposite to the direction of gravity. In Figure 2, a schematic diagram of the upward UF apparatus is illustrated. After a batch filtration cell with an effective membrane area of 12.6 cm^2^ was placed in the given direction on the angled plate, a constant pressure *p* of 98 kPa, controlled by a reducing valve, was exerted on the filtration cell by applying compressed nitrogen gas. The filtrate was collected in a conical flask placed on an electronic balance connected to a personal computer to collect and record mass *versus* time data. The weights were converted to volumes using density correlations. The filtration rate was obtained by numerical differentiation of the volume *versus* time data. Regenerated cellulose membranes with a nominal molecular weight cut-off of 10,000 Da, supplied by Millipore Corporation, were employed for all UF experiments to ensure complete rejection of the particles.

## 3. Results and Discussion

### 3.1. Comparison of Filtration Behaviors between Downward and Upward UF

**Figure 3:**
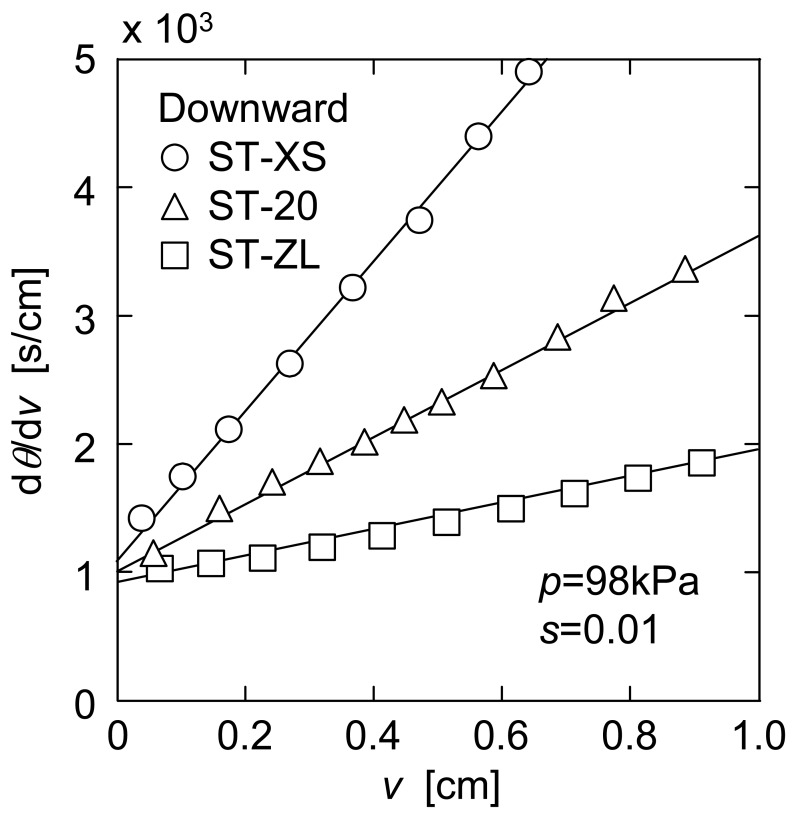
Reciprocal filtration rate in downward UF.

In Figure 3, the typical data of the flux behaviors in downward UF of three kinds of nanoparticle suspensions are shown in the form of the reciprocal filtration rate (d*θ*/d*v*) *versus* the cumulative filtrate volume *v* per unit effective membrane area. This plot is well known as the Ruth plot [4] in the classical cake filtration. In conventional downward filtration, without the filter cake exfoliation, the filtration rate declines gradually due to the build-up of a particulate cake with progression of filtration. Therefore, the plots in Figure 3 appeared to be virtually linear throughout the course of filtration in accordance with the Ruth filtration rate equation described by [4]

(1) $$\frac{\text{d}\theta }{\text{d}\nu }=\frac{\mu \alpha_{\text{av}}\rho s}{p(1-ms)}\nu +{\left(\frac{\text{d}\theta }{\text{d}\nu }\right)}_{\text{m}}$$

where *μ* is the viscosity of the filtrate, *α*_av_ is the average specific filtration resistance, *ρ* is the density of the filtrate, *s* is the mass fraction of the particles in the suspension, *p* is the applied filtration pressure, *m* is the ratio of wet to dry cake mass, and (d*θ*/d*ν*)_m_ is the reciprocal filtration rate at the start of filtration, equivalent to the flow resistance of the membrane. It is believed that these plots are still increasing linearly even if *ν* becomes larger beyond the range of the figure because the filter cake continues to grow. Also, the figure clearly demonstrates that the slope of the plot becomes steeper, *i.e.* the filtration rate becomes lower as the particle size becomes smaller. According to the Kozeny-Carman equation, the local specific filtration resistance *α* in each cross-section of compressive filter cake layer is expressed as [5]

(2) $$\alpha =\frac{180(1-ɛ)}{\rho_{\text{p}}d_{\text{s}}^{2}{ɛ}^{3}}$$

where *ε* the local porosity of compressive filter cake, and *ρ*_p_ is the density of the particle. This equation means that *α* is inversely proportional to the square of the particle diameter *d*_s_ when *ε* is the same. Although this equation also means that *α* is strongly affected by *ε*, the dependence on *d*_s_ is considered to be more dominant in this study since nanoparticles with quite different sizes were used. Thus, the filter cake with higher specific filtration resistance is formed when the smaller particles are filtered, resulting in significant decrease of the filterability.

**Figure 4:**
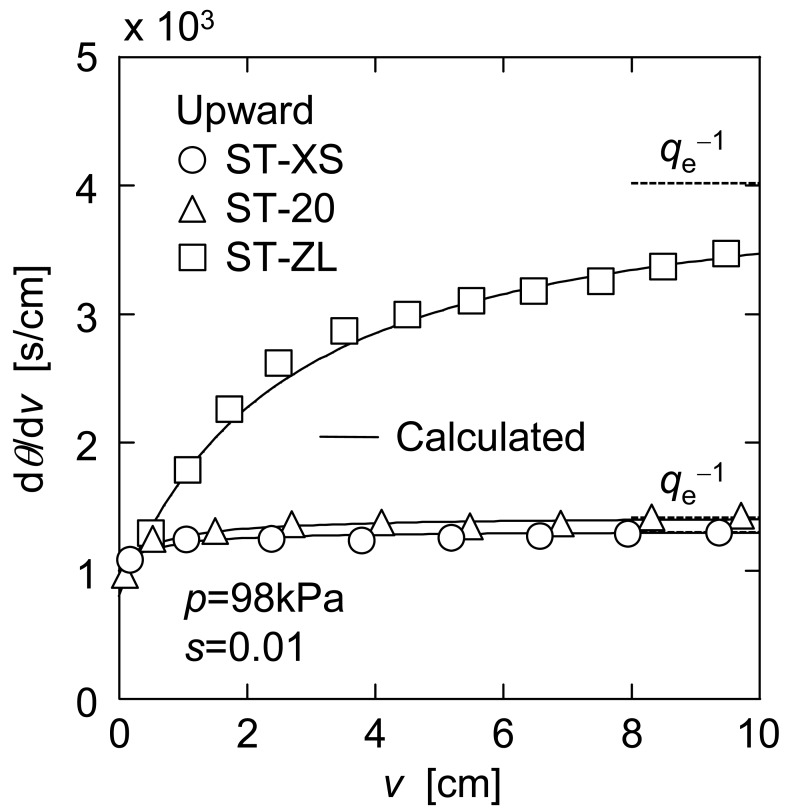
Reciprocal filtration rate in upward UF.

In Figure 4, the plots of d*θ*/d*ν versus ν* in upward UF are shown for three kinds of nanoparticle suspensions. In upward UF, a decline of the filtration rate was suppressed because the filter cake was exfoliated continuously by the effect of gravity, and as a result the reciprocal filtration rate showed a tendency to approach a steady value (d*θ*/d*ν*)_e_. Since the filter cake consisting of the micron-size particles does not exhibit the flowability in general under such a mild force as gravity, this behavior in the upward mode is one of the distinct features in membrane filtration of the nanoparticles. This figure also distinctly shows that the filtration rate increased significantly with a decrease in particle size. This may be because the filter cake consisting of smaller particles can return to the bulk suspension more easily under the influence of gravity due to the flowability caused by extremely large specific surface area. It is of great interest to note that the downward and upward modes are opposite in the dependence of the filterability of nanoparticle suspensions on the particle size.

In order to evaluate the exfoliation properties of the nanoparticle cake in upward UF, the steady filtration rate *q*_e_ (= (d*θ*/d*ν*)_e_^−1^) is used from the following section. Since some experimental data of filtration rate did not reach enough steady value during experiment, the values of *q*_e_ were determined as follows. The variable *J* is defined using the reciprocal filtration rates (d*θ*/d*ν*)_d_ and (d*θ*/d*ν*)_u_ in downward and upward modes and the reciprocal pure water flux (d*θ*/d*ν*)_m_ of the membrane as [2]

(3) $$J\equiv \frac{{\text{(d}\theta /\text{d}\nu \text{)}}_{\text{d}}-{\text{(d}\theta /\text{d}\nu \text{)}}_{\text{m}}}{{\text{(d}\theta /\text{d}\nu \text{)}}_{\text{u}}-{\text{(d}\theta /\text{d}\nu \text{)}}_{\text{m}}}$$

**Figure 5:**
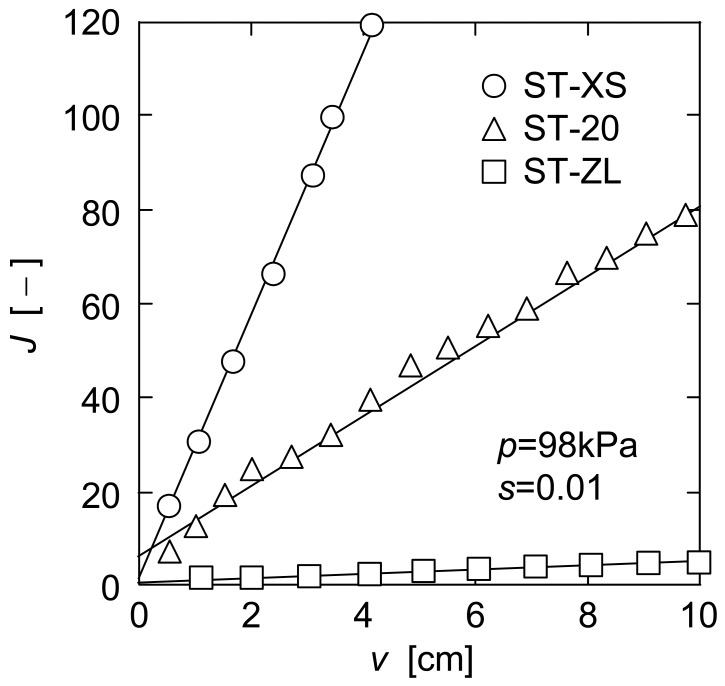
*J versus v*.

In Figure 5, *J* calculated from the data of Figure 3 and Figure 4 is plotted against the filtrate volume *ν* per unit membrane area. This figure indicates that the plot of *J versus ν* can be approximated by a straight line. Considering that (d*θ*/d*ν*)_u_ becomes *q*_e_^−1^ when *ν* is infinity, *q*_e_ is represented as the following equation using the slope *σ*_d_ of a straight line in Figure 3 and the slope *σ*_j_ of a straight line in Figure 5.

(4) $$\frac{1}{q_{\text{e}}}=\frac{\sigma_{\text{d}}}{\sigma_{\text{j}}}+{\left(\frac{\text{d}\theta }{\text{d}\nu }\right)}_{\text{m}}$$

The values of *q*_e_ obtained in this manner are depicted by the dashed lines in Figure 4. In addition, the solid lines in Figure 4 indicate the calculations based on the approximate straight lines in Figure 5.

### 3.2. Effect of Suspension Conditions on Steady Filtration Rate in Upward UF

**Figure 6:**
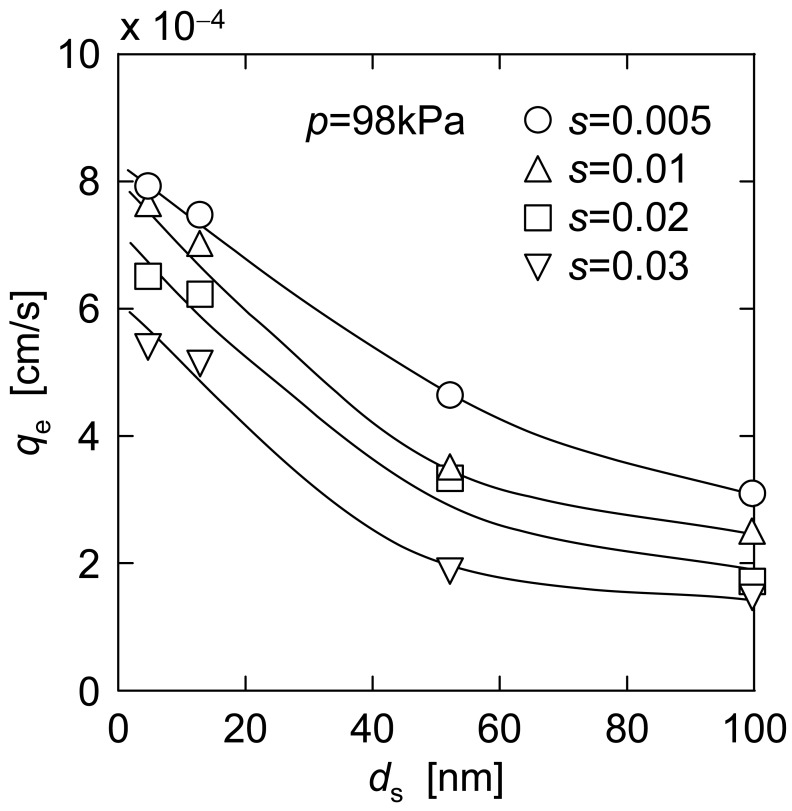
Effect of particle diameter on steady filtration rate.

**Figure 7:**
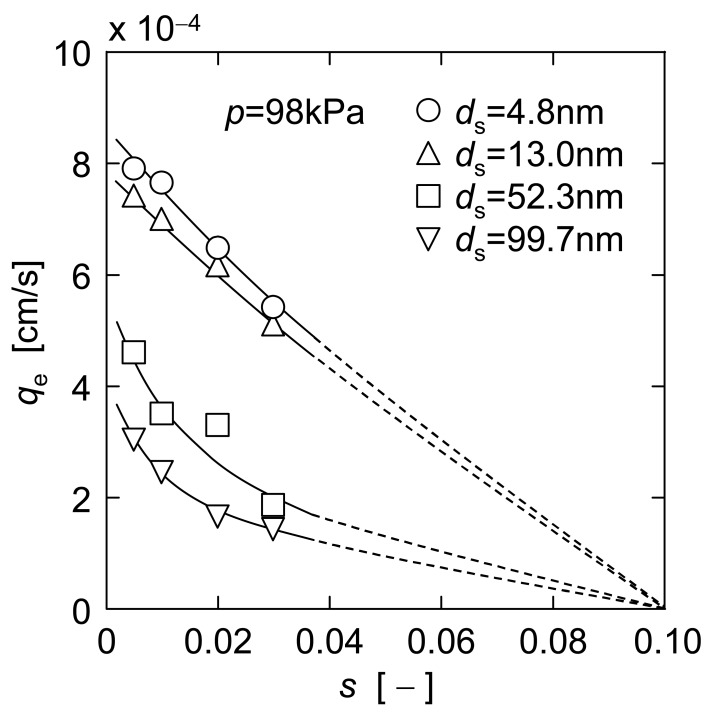
Effect of particle concentration on steady filtration rate.

The experimental data of steady filtration rate *q*_e_ in upward UF were obtained using single component suspensions of ST-XS, ST-20 and ST-ZL, and binary mixtures of ST-XS and ST-ZL, with various concentrations *s* (*s*_XS_, *s*_20_, *s*_ZL_, or *s*_XS_ + *s*_ZL_). The *q*_e_ data are plotted against the surface mean diameter *d*_s_ of particles in Figure 6 and against the particle concentration *s* in Figure 7, respectively. The surface mean diameter *d*_s_ of a binary mixture of small and large particles is calculated from the mean diameters *d*_1_ and *d*_2_ and the mass concentrations *s*_1_ and *s*_2_ of the small particle (subscript 1) and large one (subscript 2) by

(5) $$d_{\text{s}}=\frac{(s_{1}+s_{2})d_{1}d_{2}}{s_{1}d_{2}+s_{2}d_{1}}$$

For ST-XS/ST-ZL mixtures in Figure 6 and Figure 7, *d*_s_ is 52.3 nm since the concentration ratio *s*_ZL_/*s*_XS_ was adjusted to 20.8. As shown in Figure 6, the plots of *q*_e_ monotonically decreased with an increase of *d*_s_ under the condition of constant total particle concentration. And also, the *q*_e_ values in Figure 7 tended to decrease with an increase of *s* when the same particles were filtered, and it was revealed that the higher the particle concentration is, the more difficult the filter cake exfoliation becomes. This may be because the cake exfoliation is induced by the density difference between the filter cake and the bulk suspension. The behavior of plots in Figure 7 indicates the presence of the critical concentration s* that *q*_e_ becomes zero due to no cake exfoliation. It is thought that the density difference is too small to induce the cake exfoliation under the condition of the critical concentration s*. Here, the value of s* is considered as 0.1 for every colloidal silica suspension from extrapolation lines.

As mentioned above, the steady filtration rate *q*_e_ is highly dependent on the particle diameter *d*_s_ and the concentration *s*. Now, it is assumed that the estimation equation to comprehensively describe these influences can be expressed by an exponential form $q_{\text{e}}=ad_{\text{s}}^{b}{\left(s*-s\right)}^{c}$. The multiple regression analysis was applied on the basis of the experimental data in Figure 6 and Figure 7 to determine the constants *a*, *b* and *c*, and then the following estimation equation was obtained.

(6) $$\log q_{\text{e}}=-0.94-0.42\log d_{\text{s}}+1.74\log(0.1-s)$$

**Figure 8:**
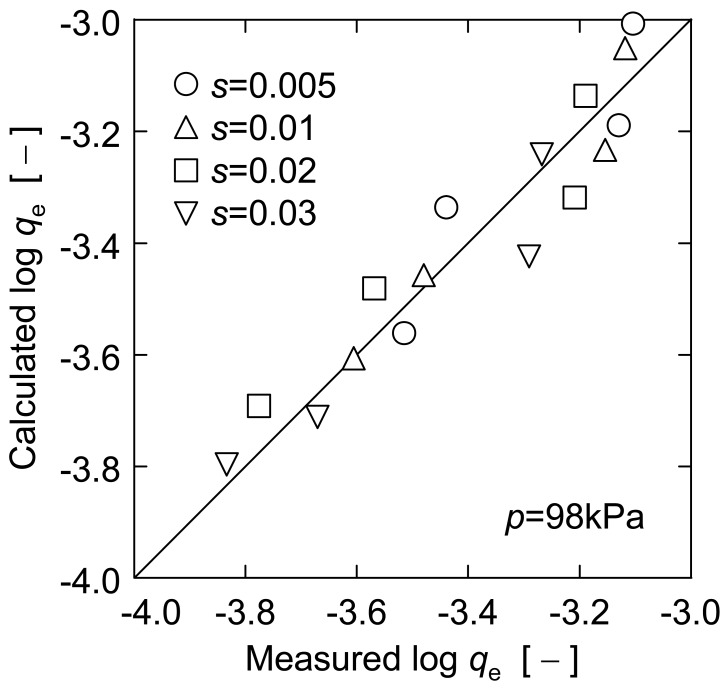
Comparison between calculated and measured values.

Figure 8 compares the calculated *q*_e_ values based on Equation (6) with the measured ones in Figure 6 and Figure 7. This figure indicates that there is generally good correlation between both data, and therefore Equation (6) would be appropriate as an estimation equation of upward filtration rate observed for a broad range of particle diameters and concentrations.

### 3.3. Effect of Addition of Small Particles on Exfoliation Properties of Binary Nanoparticle Cake

In the previous section, either dependence on the particle diameter or the concentration has been investigated. In this section, moreover, the influence of these conditions on the steady filtration rate is explored in case both mean particle diameter and particle concentration are simultaneously altered. For example, it includes the case where small particles are added into a given concentration of large particle suspension. In this operation, the addition of small particles leads to both a decrease in mean particle diameter *d*_s_ and an increase in total particle concentration *s*. This fits, for example, the operation that the nanoparticles are added into the particulate suspension as a filter aid, because the filtration rate is markedly increased as the mean diameter of particles included in the suspension becomes smaller, as shown in Figure 6, and therefore the nanoparticles may be available as a filter aid for improvement of the filtration rate in upward or dynamic filtration.

**Figure 9:**
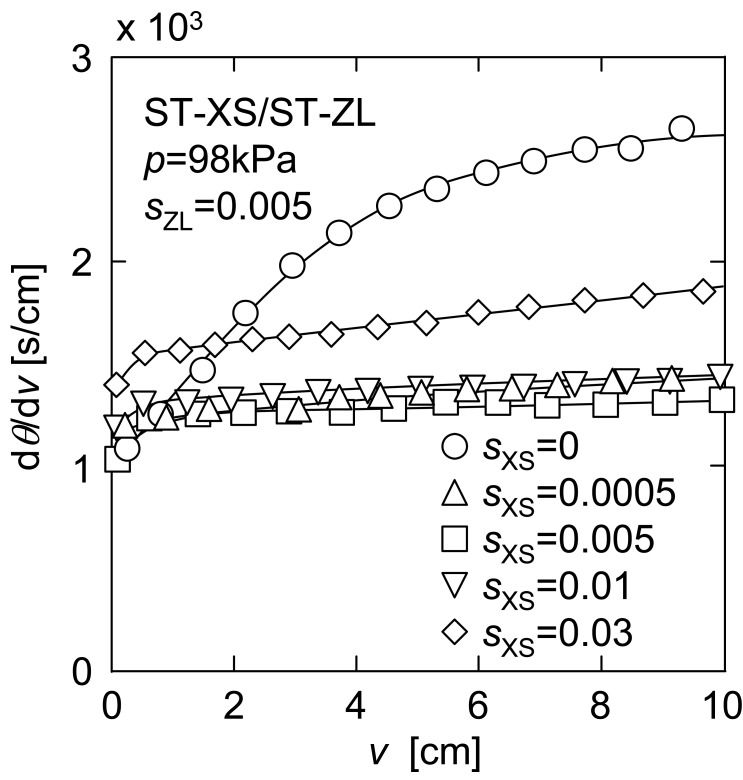
Effect of small particle concentration on filtration behavior in upward UF.

**Figure 10:**
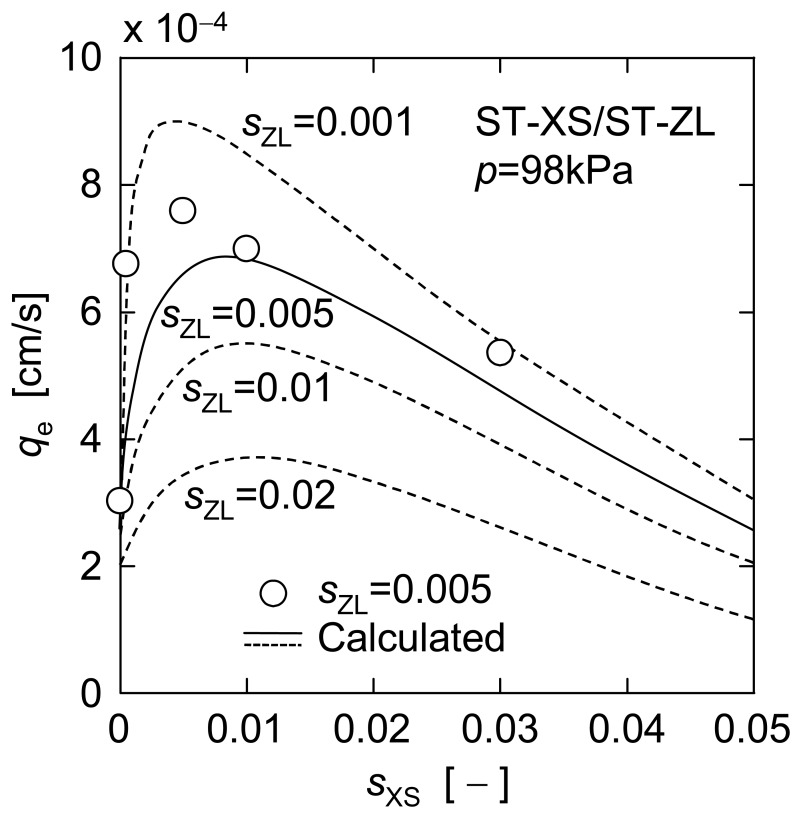
Effect of small particle concentration on steady filtration rate.

Figure 9 shows d*θ*/d*ν versus ν* in upward UF of the mixtures of a constant concentration of larger ST-ZL particle (*s*_ZL_ = 0.005) and various concentrations of smaller ST-XS particle (*s*_XS_ = 0 to 0.03). The filtration rate was markedly increased by the addition of ST-XS particles in the range of small ST-XS concentration of 0 to 0.005, while the filtration rate showed a tendency to decrease gradually with an increase in ST-XS concentration in *s*_XS_ range of more than 0.005. It is considered that the former was caused by the reduction in mean diameter, and the latter was due to the effect of the increase in total particle concentration. Figure 10 depicts the relationship between the steady filtration rate *q*_e_ estimated from the data in Figure 9 and ST-XS concentration *s*_XS_. The plot of measured values shows a definite maximum around *s*_XS_ of 0.005. Thus, the upward filtration rate in this operation is determined by a balance between contributions of the mean particle diameter and the concentration to the filter cake exfoliation.

**Figure 11:**
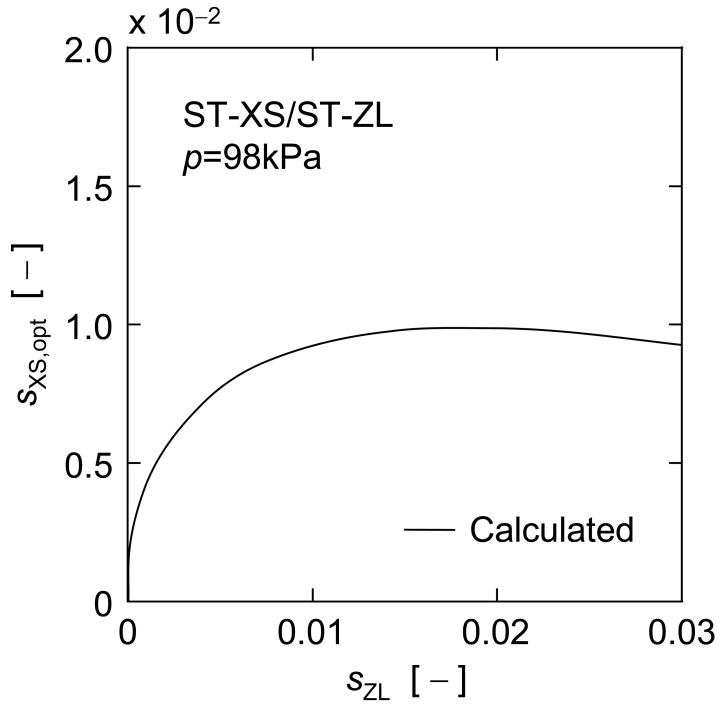
Optimal concentration of small particles for high filtration rate.

**Figure 12:**
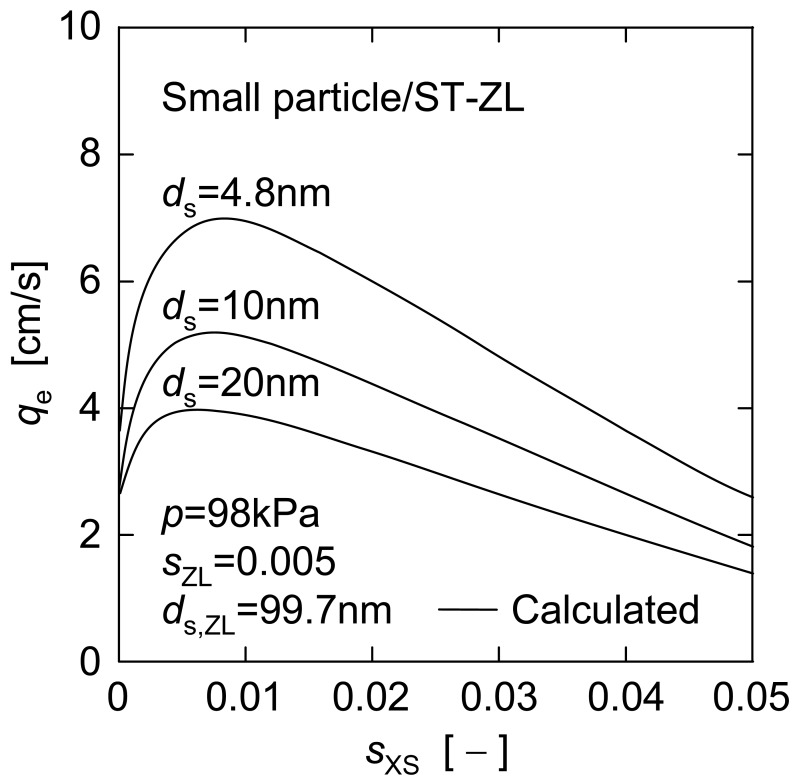
Relations between steady filtration rate and small particle concentration for different small particle diameters.

Next, the applicability of the estimation equation is focused on. In Figure 10, the calculated value based on Equation (6) is indicated by a solid line as well as the measured one. Although the estimation equation underestimated *q*_e_ value a little, it could describe the overall trend of measured values roughly, which demonstrates the effectiveness of this estimation equation. In addition, the predictions of *q*_e_ *versus s*_XS_ for other ST-ZL concentrations (*s*_ZL_ = 0.001, 0.01 and 0.02) are also indicated by the dashed lines in Figure 10. There is a definite maximum on any predicted line, which corresponds to the optimal dosage of nanoparticles where the nanoparticles are applied as a filter aid. The optimal dosages of ST-XS particles estimated in this manner are shown against the initial concentration of ST-ZL suspensions in Figure 11. Furthermore, Figure 12 shows the effect of dosage of nanoparticles with different diameters (*d*_s_ = 4.8, 10, 20 nm) for a given concentration of ST-ZL suspension (*s*_ZL_ = 0.005) on the steady filtration rate. Thus, this estimation equation is applicable to the prediction of the steady filtration rate for various conditions of feed suspensions, and also can determine the optimal dosage of nanoparticles.

## 4. Conclusions

The UF experiments of the nanoparticle suspensions with different particle diameters were conducted in the downward and upward modes. The results clearly demonstrate that as the particle diameter becomes smaller, the filtration rate decreases in downward UF in which the filtrate flow is in the same direction as gravity, while the filtration rate increases in upward UF in which the filtrate flow is opposite to the direction of gravity. It is of great interest to note that two filtration modes show quite opposite trends. Upward UF of the nanoparticle suspensions is characterized by a steady filtration rate, resulting from the filter cake exfoliation, and the steady filtration rate is strongly affected by mean diameter and total concentration of nanoparticles in the feed suspension. When small particles are added into the large particle suspension, the steady filtration rate is significantly improved due to a decrease in the mean particle diameter. However, excessive addition reduces the filtration rate due to the increase in total particle concentration. This variation of the steady filtration rate for various conditions of feed suspensions can be explained by the exponential estimation equation proposed in this study. The coefficient and indexes in this estimation equation need to be individually determined for target suspension systems because these values are dependent on the particle characteristics such as the real density and the surface conditions.
